# Co-occurrence of Obstructive Coronary Artery Disease and Takotsubo Cardiomyopathy

**DOI:** 10.7759/cureus.59111

**Published:** 2024-04-26

**Authors:** Rotimi Awoyode, David N Ray, Joseph A Akamah, Henry Okafor

**Affiliations:** 1 Internal Medicine, Meharry Medical College, Nashville, USA; 2 Cardiology, Nashville General Hospital, Nashville, USA; 3 Cardiology, Meharry Medical College, Nashville, USA; 4 Cardiology, Vanderbilt University Medical Center, Nashville, USA

**Keywords:** non-st-segment elevation myocardial infarction (nstemi), acute coronary syndrome (acs), percutaneous coronary intervention (pci), takotsubo cardiomyopathy (ttc), coronary artery occlusion, echocardiogram (echo)

## Abstract

Takotsubo syndrome, a type of transient cardiomyopathy, is typically triggered by emotional or physical stress and exhibits symptoms like acute coronary syndrome (ACS). The condition often results in apical ballooning of the left ventricle, which can hinder the heart's ability to circulate blood throughout the body effectively. While Takotsubo syndrome does not occur alongside obstructive coronary artery disease (CAD), there are rare cases where both conditions coexist. This report details an uncommon case of Takotsubo cardiomyopathy in a 49-year-old man who had previously been in remission for rectal adenocarcinoma. He presented with atypical symptoms consistent with Takotsubo cardiomyopathy while also experiencing acute occlusion of the left circumflex artery.

## Introduction

Takotsubo cardiomyopathy is a vital syndrome to consider in suspected cases of acute coronary syndrome (ACS). This cardiomyopathy should be recognized as a potential cause of cardiac death in individuals without pre-existing heart disease [1]. The heart characteristically takes on the appearance of a Japanese octopus fishing pot, locally referred to as "Takotsubo" [1]. The first case series describing Takotsubo cardiomyopathy was published in 1991 and involved five Japanese patients [2]. Patients typically present with chest pain, ST-segment elevation on electrocardiogram (ECG), and elevated cardiac markers [3]. The clinical presentation naturally follows an emotional or physical stressor triggering signs of acute myocardial infarction, such as left ventricular dysfunction, without obstructive coronary artery stenosis or spasms [3]. However, these symptoms typically resolve within a few weeks [3]. Studies have shown that Takotsubo primarily affects women; however, this could be due to underreporting the number of male cases [4]. Recent data suggest that males comprise approximately 10% of the overall Takotsubo population and have a significantly higher inpatient mortality than female patients [4].

This is an unusual case of Takotsubo cardiomyopathy involving a middle-aged man in recovery from cancer. He presented with a non-ST-segment elevation myocardial infarction (NSTEMI). His echocardiogram revealed regional wall abnormalities characteristic of Takotsubo cardiomyopathy and plaque erosion seen on cardiac catheterization, representing both a true NSTEMI and Takotsubo simultaneously. Given the uncommon presentation of ACS, this case underscores the importance of clinicians maintaining a higher index of suspicion, not solely anchoring on NSTEMI as a diagnosis but also considering Takotsubo cardiomyopathy.

## Case presentation

History of presentation

A 49-year-old man with a history of treated rectal adenocarcinoma presented to our emergency department with chest pain of two days' duration, associated with shortness of breath, nausea, and multiple episodes of vomiting over 36 hours. Two days prior to arrival, he experienced chest pain at home. A family member checked his vitals, which revealed bradycardia at 30 beats per minute (bpm). Emergency medical services were called, but the patient refused to go to the ER. 

The patient later presented to the hospital for a scheduled CT scan and was subsequently sent to the ER due to ongoing chest pain. At that time, his initial vital signs showed a blood pressure of 104/89 mmHg, a heart rate of 112 bpm, a respiratory rate of 18 breaths/min, an oxygen saturation of 98% on room air, and a temperature of 98.9°F. Physical examination was notable for chronic ill-appearance, a colostomy bag in the left lower quadrant containing brown stool, a regular heart rate and rhythm, equal breath sounds without crackles or wheezes, warm extremities, and no peripheral edema.

Past medical history

The patient's medical history includes gastroparesis, previous treatment for rectal adenocarcinoma using 5-fluorouracil, radiotherapy, and abdominopelvic resection with an end ostomy. Unfortunately, the end ostomy caused bladder injury and chronic prostatitis. However, no new cancer development has been indicated in the past one and a half years. Additionally, the patient has a history of depression and anxiety. The patient also has a family history of first-degree relatives with cancers: his father had colon cancer, his mother had uterine cancer, and his sister had pancreatic cancer. There was no family history of premature coronary artery disease (CAD).

Investigations

**Figure 1:**
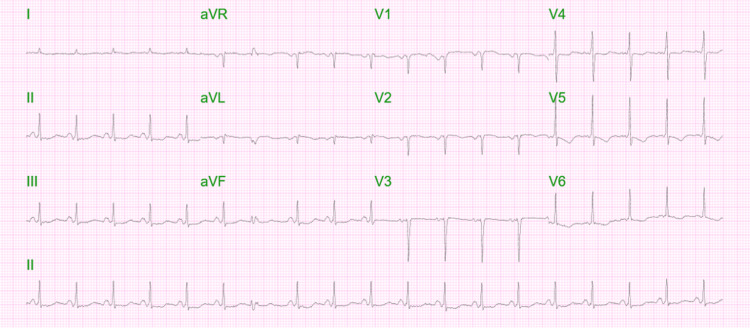
Initial ECG revealing sinus tachycardia at 120 bpm, poor R-wave progression, and inverted T-waves in leads V4-V6 without ST-segment depression or elevation. ECG: electrocardiogram

On arrival, the ECG revealed sinus tachycardia at 120 bpm, poor R-wave progression, and inverted T-waves in leads V4-V6 without ST-segment depression or elevation (Figure 1). 

Cardiac enzyme values trended every six hours as shown in Table 1. Notably, there were leukocytosis (WBC of 14.2) and a B-type natriuretic peptide (BNP) greater than 35,000 without clinical signs and symptoms of heart failure.

**Table 1:** Trending cardiac enzyme values on arrival and six hours after are shown in the table. CK: creatinine kinase; CK-MB: creatinine kinase myocardial band; ng/mL: nanograms per milliliter; U/I: units of enzyme activity per liter of serum; ug/L: micrograms per liter

	Normal value range	First blood draw	Six hours after the first blood draw	Six hours after the second blood draw
Troponin I (ng/mL)	0.00-0.056	9.900	8.883	5.220
CK (U/I)	39-308	573	357	221
CK-MB (ug/L)	0.0-3.6	47.4	15.7	8.6

A 2D transthoracic echocardiogram (TTE) demonstrated severely depressed left ventricular systolic function with marked hypokinesis of the mid, apical, anteroseptal, and inferoapical segment walls in a non-vascular pattern along with preserved contractility in the basal walls consistent with stress-induced cardiomyopathy. The estimated left ventricular ejection fraction (EF) was 15-20% (Video 1 and Video 2). 

**Video 1:** Initial echocardiogram in two-chamber view.

**Video 2:** Initial echocardiogram in four-chamber view. Videos 1a, 1b show diffuse hypokinesis in a non-vascular pattern with preserved basal wall contractility.

**Figure 2:**
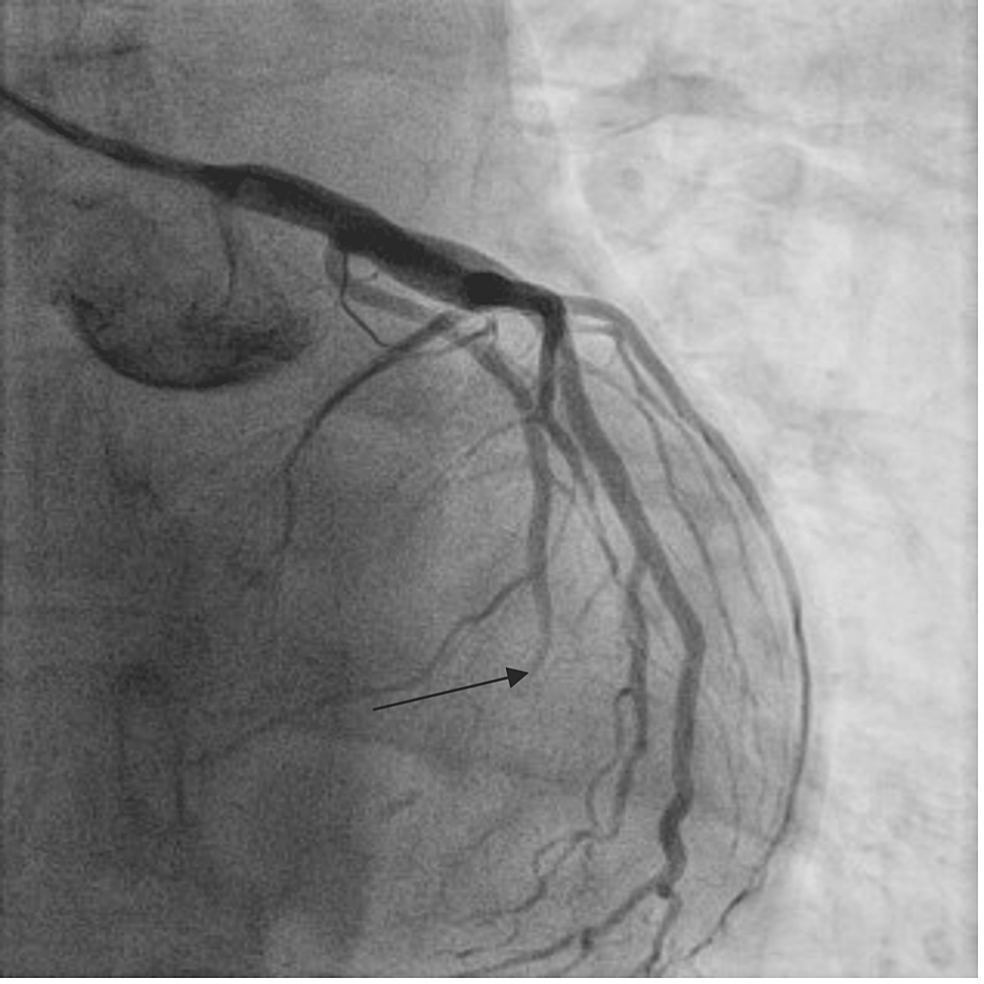
Coronary angiogram showing 100% obstruction in the mid-left circumflex artery.

**Figure 3:**
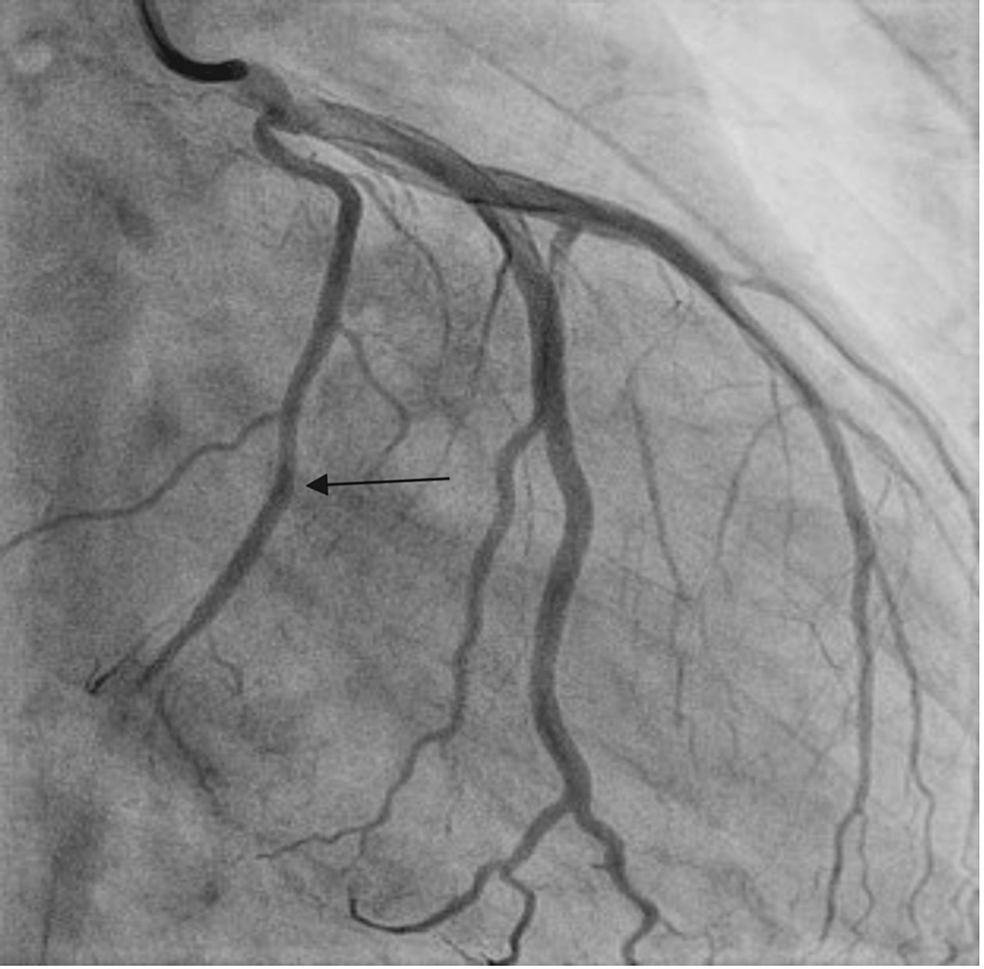
Coronary angiogram showing recanalized mid-left circumflex artery.

A left heart catheterization revealed mid-left circumflex artery severe disease (100% occlusion) with TIMI 0 flow and small thrombus burden, likely due to plaque erosion (Figure 2). The rest of the vessels had minor luminal irregularities. The affected portion was stented with a 2.5x15 mm Xience drug-eluting stent (Figure 3). 

Management

The patient was admitted to the cardiac/coronary care unit and received medical treatment for ACS, which included dual antiplatelet therapy and anticoagulation through a continuous intravenous heparin drip. Morphine and nitroglycerin helped alleviate the patient's chest pain. Every six hours, the patient underwent repeated ECG and cardiac enzyme tests, including troponins, which showed a downward trend during the admission. The TTE findings indicated Takotsubo syndrome. Subsequently, the patient underwent left heart catheterization and percutaneous intervention (PCI), with a drug-eluting stent placed in the occluded mid-left circumflex artery.

Following PCI, he was maintained on metoprolol tartrate 12.5 milligrams (mg) twice a day, aspirin 81 mg, clopidogrel 75 mg, atorvastatin 80 mg, and lisinopril 5 mg. The patient was later transferred to the telemetry step-down unit and discharged home.

The patient has been doing well since leaving the hospital. A follow-up TTE conducted 3.5 months after being diagnosed with Takotsubo cardiomyopathy showed that his left ventricular systolic function was preserved at 45%, compared to the previous 15-20%. His left ventricular wall motion, diastolic function, left ventricular filling pressure, right ventricular size, systolic function, and right atrial filling pressure were all within normal ranges (Video 3 and Video 4). 

**Video 3:** Echocardiogram in two-chamber view performed on 3.5-month follow-up.

**Video 4:** Echocardiogram in four-chamber view performed on 3.5-month follow-up. Videos 2a, 2b show the return of normal contractility in all regions.

## Discussion

When suspecting ACS, it is essential to remember Takotsubo cardiomyopathy as a potential cause of death, especially for patients without pre-existing heart disease. Takotsubo commonly occurs in postmenopausal women after exposure to unexpected emotional or physical stressors, but according to the International Takotsubo Registry study, an evident trigger is absent in 28.5% of cases [5]. This patient had a prolonged period of nausea and vomiting from gastroparesis but did not have a history of emotional stress before diagnosing Takotsubo cardiomyopathy. 

The pathobiological mechanism of Takotsubo is unknown, but most literature attributes it to excess catecholamine as the key contributor [1]. It is characterized as transient left ventricular dysfunction without obstructive coronary disease [6]. ECG features include ST-segment elevation in 43.7% and ST-segment depression in 7.7%, and other ECG findings include QT interval prolongation, T-wave inversion, abnormal Q waves, and non-specific abnormalities [5]. Serum cardiac troponin levels are elevated in most patients with Takotsubo cardiomyopathy (e.g., median initial troponin 7.7 times the upper limit of normal with an interquartile range of 2.2-24) [6]. BNP or N-terminal pro-BNP levels are also elevated in most patients diagnosed with stress cardiomyopathy [1]. 

A patient presenting to the ER with acute chest pain should be investigated for life-threatening causes, including ACS. This patient had evidence of both Takotsubo and obstructive CAD with a peak recorded cardiac troponin I at 9.9 ng/ml (normal value 0.00-0.056 ng/ml). There was 100% obstruction in the mid-left circumflex artery, but the ECG did not reveal ST-elevation myocardial infarction. One of the four proposed Mayo Clinic diagnostic criteria of Takotsubo includes the absence of obstructive coronary disease or angiographic evidence of acute plaque rupture unless there is a concurrent CAD [7]. In the International Takotsubo Registry study, 15.3% of patients with Takotsubo cardiomyopathy had concurrent CAD detected by coronary angiography [5]. Based on the clinical findings, we believe our patient is a part of that 15.3% of patients with co-occurring coronary disease. Invasive coronary angiography is essential for evaluating CAD in high-risk patients with elevated troponin levels, which is why our patient underwent this procedure [8]. It is necessary to perform an unbiased workup despite a patient presenting with classic features of other cardiac diseases.

## Conclusions

It is important to recognize that Takotsubo cardiomyopathy can occur concurrently with occlusive CAD. In patients with CAD, the possibility of missing Takotsubo cardiomyopathy is high; hence, one should have a high level of suspicion when evaluating patients with CAD. It is imperative to note that Takotsubo can occur without any emotional stressor, as seen in this patient.
